# Supplementary material for: Modeling glioblastoma heterogeneity as a dynamic network of cell states
Source: Mol Syst Biol. 2021 Sep 16;17(9):e10105. doi: 10.15252/msb.202010105 (PMC8444284; doi:10.15252/msb.202010105)
Supplement: Supplementary file 6 — Source Data for Figure 5 [file MSB-17-e10105-s004.zip › Figure5A_sourcedata/GSEA_3017/nosigdb_stateA.GseaPreranked.1622795026485/RICHARDS_NOWAKOWSKI_SCIENCE_2017_ORG_UPREG.html]

Details for gene set RICHARDS\_NOWAKOWSKI\_SCIENCE\_2017\_ORG\_UPREG[GSEA]

|  || Dataset | state53017 |
| Phenotype | NoPhenotypeAvailable |
| Upregulated in class | na\_neg |
| GeneSet | RICHARDS\_NOWAKOWSKI\_SCIENCE\_2017\_ORG\_UPREG |
| Enrichment Score (ES) | -0.38042048 |
| Normalized Enrichment Score (NES) | -2.7582207 |
| Nominal p-value | 0.0 |
| FDR q-value | 0.0 |
| FWER p-Value | 0.0 |
Table: GSEA Results Summary

  

Fig 1: Enrichment plot: RICHARDS\_NOWAKOWSKI\_SCIENCE\_2017\_ORG\_UPREG      
 Profile of the Running ES Score & Positions of GeneSet Members on the Rank Ordered List

  

| PROBE | GENE SYMBOL | GENE\_TITLE | RANK IN GENE LIST | RANK METRIC SCORE | RUNNING ES | CORE ENRICHMENT || 1 | FABP5 |  |  | 0 | 1.759 | 0.0173 | No |
| 2 | CREM |  |  | 2 | 1.023 | 0.0261 | No |
| 3 | KIF5B |  |  | 16 | 0.687 | 0.0158 | No |
| 4 | DBI |  |  | 17 | 0.683 | 0.0226 | No |
| 5 | PPA1 |  |  | 19 | 0.668 | 0.0278 | No |
| 6 | CA12 |  |  | 24 | 0.645 | 0.0289 | No |
| 7 | PGAM1 |  |  | 35 | 0.608 | 0.0218 | No |
| 8 | VIM |  |  | 46 | 0.543 | 0.0141 | No |
| 9 | ECHS1 |  |  | 67 | 0.487 | -0.0074 | No |
| 10 | HNRNPF |  |  | 70 | 0.485 | -0.0052 | No |
| 11 | FGFBP3 |  |  | 75 | 0.477 | -0.0057 | No |
| 12 | EMP3 |  |  | 101 | 0.452 | -0.0341 | No |
| 13 | PRTFDC1 |  |  | 104 | 0.448 | -0.0323 | No |
| 14 | ZEB1 |  |  | 109 | 0.446 | -0.0331 | No |
| 15 | MICU1 |  |  | 120 | 0.429 | -0.0420 | No |
| 16 | ZYX |  |  | 136 | 0.416 | -0.0576 | No |
| 17 | ST8SIA1 |  |  | 138 | 0.414 | -0.0548 | No |
| 18 | PPP2R2D |  |  | 139 | 0.413 | -0.0507 | No |
| 19 | CTBP2 |  |  | 142 | 0.413 | -0.0493 | No |
| 20 | DNAJC1 |  |  | 151 | 0.407 | -0.0557 | No |
| 21 | S100A16 |  |  | 156 | 0.404 | -0.0570 | No |
| 22 | TNC |  |  | 159 | 0.402 | -0.0557 | No |
| 23 | CCDC6 |  |  | 172 | 0.394 | -0.0675 | No |
| 24 | CALM1 |  |  | 174 | 0.391 | -0.0650 | No |
| 25 | MRPS6 |  |  | 181 | 0.389 | -0.0690 | No |
| 26 | LPAR4 |  |  | 184 | 0.387 | -0.0678 | No |
| 27 | CCNY |  |  | 199 | 0.375 | -0.0825 | No |
| 28 | PARD3 |  |  | 200 | 0.375 | -0.0788 | No |
| 29 | UGDH |  |  | 224 | 0.363 | -0.1053 | No |
| 30 | BCAN |  |  | 276 | 0.334 | -0.1689 | No |
| 31 | SEPT9 |  |  | 284 | 0.331 | -0.1748 | No |
| 32 | MT1X |  |  | 285 | 0.331 | -0.1715 | No |
| 33 | HSPD1 |  |  | 291 | 0.328 | -0.1748 | No |
| 34 | GGCT |  |  | 298 | 0.326 | -0.1795 | No |
| 35 | MLLT10 |  |  | 311 | 0.322 | -0.1920 | No |
| 36 | HSPB1 |  |  | 312 | 0.322 | -0.1889 | No |
| 37 | SORD |  |  | 317 | 0.320 | -0.1910 | No |
| 38 | ARL3 |  |  | 318 | 0.319 | -0.1878 | No |
| 39 | SWAP70 |  |  | 323 | 0.317 | -0.1899 | No |
| 40 | IDI1 |  |  | 334 | 0.314 | -0.1999 | No |
| 41 | PEG3 |  |  | 335 | 0.314 | -0.1969 | No |
| 42 | CLIC1 |  |  | 342 | 0.311 | -0.2016 | No |
| 43 | MRPS18A |  |  | 359 | 0.306 | -0.2196 | No |
| 44 | SGPL1 |  |  | 360 | 0.306 | -0.2166 | No |
| 45 | ENKUR |  |  | 389 | 0.298 | -0.2504 | No |
| 46 | TNKS2 |  |  | 390 | 0.298 | -0.2474 | No |
| 47 | ZMYND11 |  |  | 392 | 0.297 | -0.2458 | No |
| 48 | BNIP3 |  |  | 395 | 0.296 | -0.2455 | No |
| 49 | PDZD8 |  |  | 405 | 0.291 | -0.2544 | No |
| 50 | CREB5 |  |  | 407 | 0.291 | -0.2529 | No |
| 51 | ANXA7 |  |  | 408 | 0.291 | -0.2500 | No |
| 52 | ATP13A4 |  |  | 410 | 0.290 | -0.2485 | No |
| 53 | P4HA1 |  |  | 411 | 0.290 | -0.2456 | No |
| 54 | NFIA |  |  | 413 | 0.289 | -0.2441 | No |
| 55 | DOCK1 |  |  | 414 | 0.289 | -0.2412 | No |
| 56 | SHC3 |  |  | 421 | 0.288 | -0.2463 | No |
| 57 | ACBD5 |  |  | 422 | 0.288 | -0.2434 | No |
| 58 | ZNF32 |  |  | 426 | 0.287 | -0.2445 | No |
| 59 | MIS18A |  |  | 439 | 0.284 | -0.2575 | No |
| 60 | ADAM23 |  |  | 449 | 0.281 | -0.2665 | No |
| 61 | SLITRK2 |  |  | 458 | 0.279 | -0.2742 | No |
| 62 | HSPE1 |  |  | 465 | 0.277 | -0.2794 | No |
| 63 | DNPH1 |  |  | 466 | 0.277 | -0.2766 | No |
| 64 | MT2A |  |  | 473 | 0.275 | -0.2818 | No |
| 65 | AMMECR1 |  |  | 481 | 0.274 | -0.2883 | No |
| 66 | PSAT1 |  |  | 491 | 0.271 | -0.2974 | No |
| 67 | REEP3 |  |  | 496 | 0.271 | -0.3000 | No |
| 68 | SUGT1 |  |  | 513 | 0.269 | -0.3183 | No |
| 69 | ARC |  |  | 514 | 0.269 | -0.3156 | No |
| 70 | ANAPC16 |  |  | 515 | 0.268 | -0.3130 | No |
| 71 | QDPR |  |  | 517 | 0.267 | -0.3117 | No |
| 72 | PXDN |  |  | 524 | 0.266 | -0.3169 | No |
| 73 | ZFP36L1 |  |  | 532 | 0.264 | -0.3235 | No |
| 74 | TCTN3 |  |  | 533 | 0.264 | -0.3209 | No |
| 75 | ITGA6 |  |  | 544 | 0.261 | -0.3314 | No |
| 76 | VEGFA |  |  | 546 | 0.261 | -0.3302 | No |
| 77 | DSEL |  |  | 559 | 0.258 | -0.3434 | No |
| 78 | PSMG2 |  |  | 576 | 0.254 | -0.3618 | No |
| 79 | SOX9 |  |  | 577 | 0.254 | -0.3593 | No |
| 80 | SEC23IP |  |  | 578 | 0.253 | -0.3568 | No |
| 81 | CD82 |  |  | 597 | 0.250 | -0.3780 | Yes |
| 82 | ITGAV |  |  | 599 | -0.250 | -0.3768 | Yes |
| 83 | PLEKHA5 |  |  | 600 | -0.252 | -0.3743 | Yes |
| 84 | NES |  |  | 603 | -0.252 | -0.3745 | Yes |
| 85 | CD99 |  |  | 604 | -0.252 | -0.3720 | Yes |
| 86 | SYPL1 |  |  | 608 | -0.255 | -0.3734 | Yes |
| 87 | NLGN4X |  |  | 613 | -0.259 | -0.3761 | Yes |
| 88 | ABAT |  |  | 617 | -0.259 | -0.3775 | Yes |
| 89 | FAM111A |  |  | 618 | -0.259 | -0.3749 | Yes |
| 90 | PLIN3 |  |  | 619 | -0.259 | -0.3724 | Yes |
| 91 | RGS16 |  |  | 620 | -0.260 | -0.3698 | Yes |
| 92 | AIG1 |  |  | 621 | -0.260 | -0.3672 | Yes |
| 93 | DAG1 |  |  | 622 | -0.260 | -0.3647 | Yes |
| 94 | TACC1 |  |  | 623 | -0.260 | -0.3621 | Yes |
| 95 | ARHGEF26 |  |  | 628 | -0.262 | -0.3648 | Yes |
| 96 | CHMP2A |  |  | 631 | -0.262 | -0.3648 | Yes |
| 97 | TMTC2 |  |  | 633 | -0.263 | -0.3635 | Yes |
| 98 | CAMK2D |  |  | 635 | -0.264 | -0.3623 | Yes |
| 99 | SHROOM3 |  |  | 639 | -0.264 | -0.3636 | Yes |
| 100 | GNG12 |  |  | 640 | -0.265 | -0.3610 | Yes |
| 101 | CLCN3 |  |  | 641 | -0.266 | -0.3584 | Yes |
| 102 | MAGI1 |  |  | 642 | -0.266 | -0.3557 | Yes |
| 103 | SIPA1L1 |  |  | 645 | -0.267 | -0.3557 | Yes |
| 104 | ARHGEF6 |  |  | 659 | -0.273 | -0.3701 | Yes |
| 105 | SORT1 |  |  | 664 | -0.279 | -0.3726 | Yes |
| 106 | NOTCH1 |  |  | 666 | -0.280 | -0.3711 | Yes |
| 107 | PEA15 |  |  | 668 | -0.281 | -0.3697 | Yes |
| 108 | SPRY2 |  |  | 669 | -0.282 | -0.3669 | Yes |
| 109 | CKB |  |  | 676 | -0.287 | -0.3719 | Yes |
| 110 | SEMA6A |  |  | 679 | -0.292 | -0.3717 | Yes |
| 111 | RHOJ |  |  | 682 | -0.293 | -0.3714 | Yes |
| 112 | DDR1 |  |  | 683 | -0.294 | -0.3685 | Yes |
| 113 | GPC4 |  |  | 687 | -0.295 | -0.3695 | Yes |
| 114 | RPS27L |  |  | 688 | -0.295 | -0.3666 | Yes |
| 115 | AKAP12 |  |  | 690 | -0.296 | -0.3650 | Yes |
| 116 | HS6ST1 |  |  | 692 | -0.297 | -0.3634 | Yes |
| 117 | SCOC |  |  | 693 | -0.297 | -0.3605 | Yes |
| 118 | STIM2 |  |  | 694 | -0.298 | -0.3576 | Yes |
| 119 | S100B |  |  | 696 | -0.299 | -0.3559 | Yes |
| 120 | ZFHX4 |  |  | 700 | -0.301 | -0.3569 | Yes |
| 121 | ATP6AP2 |  |  | 705 | -0.304 | -0.3591 | Yes |
| 122 | SMARCA2 |  |  | 711 | -0.310 | -0.3626 | Yes |
| 123 | ASCC3 |  |  | 718 | -0.314 | -0.3674 | Yes |
| 124 | ARAP2 |  |  | 719 | -0.315 | -0.3643 | Yes |
| 125 | FZD7 |  |  | 721 | -0.316 | -0.3625 | Yes |
| 126 | BICD1 |  |  | 723 | -0.319 | -0.3607 | Yes |
| 127 | ID2 |  |  | 727 | -0.323 | -0.3614 | Yes |
| 128 | C1orf61 |  |  | 733 | -0.328 | -0.3648 | Yes |
| 129 | ELOVL2 |  |  | 735 | -0.329 | -0.3628 | Yes |
| 130 | HES1 |  |  | 736 | -0.331 | -0.3596 | Yes |
| 131 | TIMP2 |  |  | 737 | -0.332 | -0.3563 | Yes |
| 132 | PON2 |  |  | 743 | -0.338 | -0.3595 | Yes |
| 133 | LRRCC1 |  |  | 746 | -0.341 | -0.3588 | Yes |
| 134 | MYO6 |  |  | 750 | -0.343 | -0.3593 | Yes |
| 135 | HEY1 |  |  | 751 | -0.344 | -0.3559 | Yes |
| 136 | C1orf122 |  |  | 753 | -0.345 | -0.3539 | Yes |
| 137 | SRGAP3 |  |  | 755 | -0.345 | -0.3518 | Yes |
| 138 | FZD3 |  |  | 756 | -0.345 | -0.3484 | Yes |
| 139 | CYP51A1 |  |  | 759 | -0.349 | -0.3475 | Yes |
| 140 | WSCD1 |  |  | 761 | -0.351 | -0.3454 | Yes |
| 141 | SLC4A4 |  |  | 763 | -0.355 | -0.3432 | Yes |
| 142 | BOC |  |  | 766 | -0.359 | -0.3423 | Yes |
| 143 | ALDH3A2 |  |  | 767 | -0.359 | -0.3388 | Yes |
| 144 | MSI2 |  |  | 771 | -0.361 | -0.3391 | Yes |
| 145 | ITGB8 |  |  | 774 | -0.363 | -0.3382 | Yes |
| 146 | MLC1 |  |  | 783 | -0.369 | -0.3450 | Yes |
| 147 | PIK3C2A |  |  | 787 | -0.372 | -0.3453 | Yes |
| 148 | GNAO1 |  |  | 788 | -0.373 | -0.3416 | Yes |
| 149 | TUBB2A |  |  | 792 | -0.375 | -0.3419 | Yes |
| 150 | CDH4 |  |  | 794 | -0.376 | -0.3395 | Yes |
| 151 | SQSTM1 |  |  | 795 | -0.379 | -0.3357 | Yes |
| 152 | NPC2 |  |  | 800 | -0.383 | -0.3372 | Yes |
| 153 | GPC1 |  |  | 801 | -0.384 | -0.3334 | Yes |
| 154 | MSMO1 |  |  | 803 | -0.386 | -0.3309 | Yes |
| 155 | CRIM1 |  |  | 805 | -0.388 | -0.3284 | Yes |
| 156 | CXXC5 |  |  | 809 | -0.391 | -0.3285 | Yes |
| 157 | SSX2IP |  |  | 810 | -0.392 | -0.3246 | Yes |
| 158 | PHPT1 |  |  | 811 | -0.393 | -0.3207 | Yes |
| 159 | MRC2 |  |  | 813 | -0.395 | -0.3182 | Yes |
| 160 | FHL1 |  |  | 814 | -0.397 | -0.3143 | Yes |
| 161 | LTBP1 |  |  | 815 | -0.397 | -0.3103 | Yes |
| 162 | IFI27L2 |  |  | 820 | -0.402 | -0.3116 | Yes |
| 163 | HOPX |  |  | 821 | -0.403 | -0.3077 | Yes |
| 164 | DDAH1 |  |  | 828 | -0.413 | -0.3115 | Yes |
| 165 | PAM |  |  | 832 | -0.422 | -0.3112 | Yes |
| 166 | ATF3 |  |  | 836 | -0.428 | -0.3109 | Yes |
| 167 | PLOD2 |  |  | 838 | -0.431 | -0.3080 | Yes |
| 168 | NACC2 |  |  | 839 | -0.431 | -0.3038 | Yes |
| 169 | CHPT1 |  |  | 844 | -0.437 | -0.3047 | Yes |
| 170 | SDCBP |  |  | 845 | -0.438 | -0.3004 | Yes |
| 171 | ZFP36L2 |  |  | 848 | -0.443 | -0.2986 | Yes |
| 172 | TMEM47 |  |  | 855 | -0.447 | -0.3021 | Yes |
| 173 | APC |  |  | 856 | -0.447 | -0.2977 | Yes |
| 174 | SLC1A2 |  |  | 860 | -0.450 | -0.2972 | Yes |
| 175 | SERPINB6 |  |  | 861 | -0.452 | -0.2927 | Yes |
| 176 | DOK5 |  |  | 862 | -0.457 | -0.2882 | Yes |
| 177 | EZR |  |  | 865 | -0.464 | -0.2863 | Yes |
| 178 | SLC35F1 |  |  | 871 | -0.482 | -0.2881 | Yes |
| 179 | ANXA2 |  |  | 872 | -0.482 | -0.2833 | Yes |
| 180 | MOXD1 |  |  | 878 | -0.498 | -0.2850 | Yes |
| 181 | NDFIP1 |  |  | 882 | -0.510 | -0.2839 | Yes |
| 182 | PDGFC |  |  | 883 | -0.515 | -0.2788 | Yes |
| 183 | JUN |  |  | 886 | -0.525 | -0.2763 | Yes |
| 184 | F3 |  |  | 888 | -0.526 | -0.2724 | Yes |
| 185 | CALD1 |  |  | 889 | -0.527 | -0.2672 | Yes |
| 186 | PDPN |  |  | 890 | -0.529 | -0.2620 | Yes |
| 187 | UBL3 |  |  | 893 | -0.535 | -0.2594 | Yes |
| 188 | GPC6 |  |  | 896 | -0.540 | -0.2567 | Yes |
| 189 | GPM6B |  |  | 897 | -0.542 | -0.2513 | Yes |
| 190 | ITGB5 |  |  | 903 | -0.558 | -0.2524 | Yes |
| 191 | ATP1B2 |  |  | 904 | -0.558 | -0.2469 | Yes |
| 192 | MT3 |  |  | 905 | -0.559 | -0.2414 | Yes |
| 193 | SDC2 |  |  | 908 | -0.568 | -0.2384 | Yes |
| 194 | SAT1 |  |  | 909 | -0.573 | -0.2327 | Yes |
| 195 | DKK3 |  |  | 912 | -0.579 | -0.2297 | Yes |
| 196 | ARID5B |  |  | 915 | -0.586 | -0.2265 | Yes |
| 197 | RAB31 |  |  | 916 | -0.586 | -0.2207 | Yes |
| 198 | PEG10 |  |  | 917 | -0.587 | -0.2150 | Yes |
| 199 | GLUL |  |  | 920 | -0.612 | -0.2116 | Yes |
| 200 | NEDD9 |  |  | 923 | -0.625 | -0.2080 | Yes |
| 201 | ZMAT3 |  |  | 929 | -0.650 | -0.2082 | Yes |
| 202 | TTYH1 |  |  | 932 | -0.652 | -0.2044 | Yes |
| 203 | CST3 |  |  | 933 | -0.680 | -0.1977 | Yes |
| 204 | CHN1 |  |  | 934 | -0.680 | -0.1910 | Yes |
| 205 | EFNB2 |  |  | 936 | -0.682 | -0.1856 | Yes |
| 206 | CLU |  |  | 938 | -0.685 | -0.1801 | Yes |
| 207 | HEPACAM |  |  | 943 | -0.709 | -0.1784 | Yes |
| 208 | RNF180 |  |  | 945 | -0.725 | -0.1725 | Yes |
| 209 | LRRC17 |  |  | 949 | -0.769 | -0.1689 | Yes |
| 210 | IFITM3 |  |  | 954 | -0.830 | -0.1660 | Yes |
| 211 | CD44 |  |  | 957 | -0.851 | -0.1602 | Yes |
| 212 | CNN3 |  |  | 961 | -0.883 | -0.1554 | Yes |
| 213 | PPP1CB |  |  | 962 | -0.885 | -0.1467 | Yes |
| 214 | TAGLN2 |  |  | 963 | -0.891 | -0.1379 | Yes |
| 215 | ZBTB20 |  |  | 964 | -0.895 | -0.1291 | Yes |
| 216 | LGALS3 |  |  | 970 | -0.989 | -0.1259 | Yes |
| 217 | FABP7 |  |  | 971 | -0.994 | -0.1161 | Yes |
| 218 | SLC1A3 |  |  | 973 | -1.097 | -0.1066 | Yes |
| 219 | LRRN3 |  |  | 977 | -1.233 | -0.0984 | Yes |
| 220 | CYR61 |  |  | 978 | -1.296 | -0.0857 | Yes |
| 221 | PMP22 |  |  | 982 | -1.519 | -0.0746 | Yes |
| 222 | IGFBP5 |  |  | 983 | -1.595 | -0.0589 | Yes |
| 223 | SPARCL1 |  |  | 984 | -1.810 | -0.0411 | Yes |
| 224 | NEAT1 |  |  | 986 | -2.049 | -0.0222 | Yes |
| 225 | GFAP |  |  | 987 | -2.255 | -0.0000 | Yes |
Table: GSEA details [plain text format]

  

Fig 2: RICHARDS\_NOWAKOWSKI\_SCIENCE\_2017\_ORG\_UPREG: Random ES distribution      
 Gene set null distribution of ES for **RICHARDS\_NOWAKOWSKI\_SCIENCE\_2017\_ORG\_UPREG**
